# The pandemic within the pandemic: the surge of neuropsychological disorders in Italian children during the COVID-19 era

**DOI:** 10.1186/s13052-022-01324-4

**Published:** 2022-07-27

**Authors:** Elena Bozzola, Pietro Ferrara, Giulia Spina, Alberto Villani, Marco Roversi, Massimiliano Raponi, Giovanni Corsello, Annamaria Staiano, the Italian Pediatric COVID-19 Board, Francesco Chiarelli, Federica Cavallo, Giovanni Farello, Nadia Rossi, Carmela Salladini, Sergio Manieri, MariaPia Mirauda, Giacomo Biasucci, Andrea Cella, Gianluca Vergine, Angela Troisi, Federico Marchetti, Enrico Valletta, Marcello Stella, Marcello Lanari, Duccio Maria Cordelli, Ilaria Corsini, Jacopo Pruccoli, Chiara Ghizzi, Chiara Franzonello, Egidio Barbi, Alessandro Amaddeo, Ilaria Liguoro, Paola Cogo, Giuliana Morabito, Maria Rosaria Marchili, Carla Brusco, Cristina Mascolo, Riccardo Borea, Emanuela Piccotti, Tommaso Bellini, Carlo Agostoni, Raffaele Badolato, Camilla Dallavilla, Leonardo Felici, Simone Mattozzi, Guido Pennoni, Elisabetta Mencaroni

**Affiliations:** The Italian Pediatric Society, 00100 Rome, Italy

**Keywords:** COVID19, Neuropsychological disorders, Children

## Abstract

**Background:**

Quarantine and isolation measures during COVID-19 pandemic may have caused additional stress and challenged the mental health of the youth. Aim of the study is to investigate the COVID-19 pandemic impact on neuropsychological disorders (NPD) of Italian children and adolescents to provide general pediatric recommendations.

**Material and methods:**

A retrospective multicenter observational study was planned by the Italian Pediatric Society (SIP) to explore the impact of COVID-19 on the access of children to pediatric Emergency Departments (pED) for the evaluation of neuropsychological symptoms, collecting the classification codes of diagnoses between March 1, 2019 and March 2, 2021. The period study was split into two sub-periods: a pre COVID-19 period (from March 1 2019 to March 1, 2020) and a COVID-19 period (from March 2, 2020 to March 2, 2021). As additional information, data on NPD hospitalizations in any pediatric department of the involved centers were recorded.

**Results:**

During the study period, a total of 533,318 children were admitted to the pED involved in the study. Despite a 48.2% decline of pED admissions, there was a significant increase (83.1%) in patient admissions for NPD. The most frequent NPD conditions which increased during the COVID-19 pandemic were suicidal ideation (+ 147%), depression (+ 115%), eating disorder (+ 78.4%), and psychosis (+ 17.2%). During the pandemic period, a 39.5% increase in NPD hospitalizations was observed as well. The NPD disorders that mostly required hospitalizations were suicidal ideation (+ 134%), depression (+ 41.4%), eating disorder (+ 31.4%), and drug abuse (+ 26.7%).

COVID-19 pandemic had a major impact on children's health, mainly on their NPD development. Neuropsychological assessment should be required at the primary level, in the pediatrician's office, to facilitate early capture of the sign of impairment and provide an adequate treatment.

**Conclusion:**

SIP underlines the psychological consequences of COVID 19 pandemic on the youngest and recommends an early identification of NPD in the pediatric population to avoid other serious consequences for children's physical and mental health.

**Supplementary Information:**

The online version contains supplementary material available at 10.1186/s13052-022-01324-4.

## Background

On January 30, 2020, the first cases of COVID-19 were registered in Italy. Thereafter, Italy became the first European country to be severely affected by the SARS-CoV-2 pandemic. After the rapid and uncontrollable spread of the disease, the Italian government was forced to implement a countrywide quarantine on March 9, 2020 and public health measures to prevent the spread of COVID-19. Such measures included restrictions of movement, childcare and school closures, discontinuation of after-school activities, playground and public park closure, quarantine, and isolation of suspected and confirmed cases. In this pivotal time, access to the internet drastically increased, as social contacts, school lessons, and online medical consultation were transferred online. Emerging evidence suggests that prolonged school closure, home confinement and social restrictions during disease outbreak could have serious consequences for children's physical and mental health [1–5]. According to a recent WHO report, 10 to 20 percent of children and adolescents worldwide suffer from mental disorders. In more detail, a worldwide shift in children's behavior and perceptions of their future has been described, with 46% of them being less motivated to do normal daily activities. In addition, neuropsychiatric disorders have become the leading cause of disability in youth, and suicide has become the second leading cause of death among youth ages 15–29 [6, 7].

The aim of this study is to investigate the COVID-19 pandemic impact on neuropsychological disorders (NPD) of Italian children and adolescents, to provide general pediatric recommendations, and to avoid other serious consequences for children's physical and mental health.

## Material and methods

A retrospective multicenter observational study was planned by the Italian Pediatric Society (SIP) to explore the impact of COVID-19 on the access of children to pediatric Emergency Departments (pED) for the evaluation of neuropsychological symptoms. The 19 Regional Presidents of SIP were invited to participate with at least one pED. Data regarding the total number of pediatric accesses for any pathology and those for NPD at the pED were collected between March 1, 2019 and March 2, 2021. As additional information, data on neuropsychiatric hospitalizations in any pediatric department of the involved centers were recorded. A Regional Coordinating Center collected data from each Italian pED according to International Classification Codes (ICD) classification codes of diagnoses, which is used to register patients diagnoses at the hospitals of the Italian National Health Service. All patients with diagnosis of neuropsychological disorder at discharge from the pED or department were included. NPDs were described as follows: suicidal ideation, psychosis, neurotic disorders, eating disorders, depression, anxiety, and drug/alcohol abuse, corresponding to the following ICD-9 codes: psychosis (codes 291–299), neurotic and non-psychotic disorders (codes 300–314), suicidal ideation (code V62.84), and eating disorders (307). Diagnostic and Statistical Manual of Mental Disorders 5th edn, Section II, serve clinicians as a guide to identify the most prominent symptoms that should be assessed when diagnosing a disorder and assist the physician in the classification of the disorders mainly considering diagnostic criteria. According to the DSM-5 classification, these codes included patients with schizophrenia spectrum and other psychotic disorders; bipolar and related disorders; anxiety disorders; obsessive–compulsive and related disorders; trauma and stressor-related disorders; dissociative disorders; somatic symptom and related disorders; substance related and addictive disorders; depressive disorders, including major depressive disorder; personality disorders including borderline personality disorder; eating disorders including anorexia nervosa, bulimia nervosa, binge eating disorder, avoidant/restrictive food intake disorder (ARFID) [8]. Patients older than 18 years and/or accessing the emergency department more than once in a month for a NPD were excluded.

The period study was divided into the following two sub-periods:

1. Pre COVID-19 period, from March 1 2019 to March 1, 2020
2. COVID-19 period, from March 2, 2020 to March 2, 2021.

### Statistical analysis

The characteristics of the sample were reported as absolute numbers and proportions with the total national and regional cases at the denominator. The comparison between the pre COVID-19 and COVID-19 period, for each category, was made by relative risk calculation. The percentage change between the two periods was compared between the various regions by the Z-test.

The software R, version 3.2.3 (R Foundation for Statistical Computing, Vienna, Austria. http://www.R-project.org/) was used for data analysis. A *p*-value less than 0.05 was considered statistically significant.

## Results

Nine Italian regions agreed to participate in the study. In detail, Emilia Romagna, Friuli Venezia Giulia, Lombardia, Liguria, Marche, Umbria Abruzzo, Lazio, and Basilicata entered the study. Enrolled centers are reported as Supplementary Material.

The number of children who accessed pEDs and NPDs diagnoses during the study period are reported in Table 1.

**Table 1:** Pediatric admissions to EDs in the pre COVID-19 and COVID-19 periods

	pre COVID-19	COVID-19	% change	*p*-value
All regions				
Total cases	351,559	181,759	-	-
NPD	2369 (0.67%)	2242 (1.23%)	+ 83.1%	< 0.001
Suicidal ideation	18 (0.76%)	42 (1.87%)	+ 147%	0.449
Psychosis	338 (14.3%)	375 (16.7%)	+ 17.2%	0.054
Neurosis	2132 (89.9%)	1954 (87.2%)	-3.16%	0.095
ED	212 (8.9%)	358 (15.9%)	+ 78.4%	< 0.001
Depression	51 (2.15%)	104 (4.64%)	+ 115%	0.092
Anxiety	705 (29.8%)	548 (24.4%)	-17.9%	< 0.001
Alcohol/drug abuse	191 (8.06%)	158 (7.05%)	-12.6%	0.491
**Abruzzo**				
Total cases	34,630	13,904	-	-
NPD	254 (0.73%)	142 (1.02%)	+ 40%	0.566
Suicidal ideation	2 (7.9%)	0 (0%)	/	0.881
Psychosis	42 (16.5%)	17 (11.9%)	-27.6%	0.383
Neurosis	210 (82.7%)	124 (87.3%)	+ 5.62%	0.375
ED	0 (0%)	6 (4.23%)	/	0.419
Depression	1 (3.94%)	0 (0%)	/	0.940
Anxiety	112 (44.1%)	60 (42.3%)	-4.18%	0.725
Alcohol/drug abuse	43 (16.9%)	21 (14.8%)	-12.6%	0.409
**Basilicata**				
Total cases	4250	2073	-	-
NPD	29 (0.68%)	16 (0.77%)	+ 13.1%	0.947
Suicidal ideation	0 (0%)	0 (0%)	/	1.000
Psychosis	0 (0%)	0 (0%)	/	1.000
Neurosis	21 (72.4%)	12 (75.0%)	+ 3.57%	0.966
ED	0 (0%)	0 (0%)	/	1.000
Depression	0 (0%)	0 (0%)	/	1.000
Anxiety	0 (0%)	0 (0%)	/	1.000
Alcohol/drug abuse	0 (0%)	0 (0%)	/	1.000
**Emilia Romagna**				
Total cases	57,495	25,219	-	-
NPD	113 (0.20%)	106 (0.42%)	+ 110%	0.554
Suicidal ideation	0 (0%)	0 (0%)	/	1.000
Psychosis	24 (21.2%)	21 (19.8%)	-6.72%	0.833
Neurosis	89 (78.8%)	85 (80.2%)	+ 1.81%	0.833
ED	1 (0.9%)	2 (1.9%)	+ 113.2%	0.882
Depression	5 (4.42%)	5 (4.72%)	+ 6.60%	0.966
Anxiety	0 (0%)	1 (0.94%)	/	0.889
Alcohol/drug abuse	7 (6.19%)	22 (20.8%)	+ 235.0%	0.031
**Friuli Venezia Giulia**				
Total cases	64,485	25,101	-	-
NPD	557 (0.86%)	366 (1.46%)	+ 68.8%	0.110
Suicidal ideation	6 (1.08%)	12 (3.28%)	+ 204.8%	0.513
Psychosis	24 (4.31%)	19 (5.19%)	+ 20.5%	0.793
Neurosis	527 (94.6%)	335 (91.5%)	-3.26%	0.359
ED	20 (3.59%)	21 (5.74%)	+ 59.8%	0.523
Depression	4 (0.72%)	1 (0.27%)	-61.9%	0.895
Anxiety	302 (54.2%)	193 (52.7%)	-2.74%	0.659
Alcohol/drug abuse	72 (12.9%)	38 (10.4%)	-19.7%	0.449
**Lazio**				
Total cases	57,980	39,673		
NPD	926 (1.6%)	1312 (3.31%)	+ 107.1%	< 0.001
Suicidal ideation	2 (0.22%)	19 (1.45%)	+ 570.5%	0.566
Psychosis	196 (21.2%)	265 (20.2%)	-4.57%	0.652
Neurosis	728 (78.6%)	1025 (78.1%)	-0.63%	0.818
ED	177 (19.1%)	301 (22.9%)	+ 20.0%	0.745
Depression	38 (4.10%)	88 (6.71%)	+ 63.4%	0.225
Anxiety	180 (19.4%)	211 (16.1%)	-17.3%	0.118
Alcohol/drug abuse	23 (2.48%)	24 (1.83%)	-26.4%	0.760
**Liguria**				
Total cases	37,237	20,042	-	-
NPD	96 (0.26%)	54 (0.27%)	+ 4.51%	0.979
Suicidal ideation	0 (0%)	0 (0%)	/	1.000
Psychosis	14 (14.6%)	10 (18.5%)	+ 26.9%	0.645
Neurosis	81 (84.4%)	42 (77.8%)	-7.82%	0.438
ED	0 (0%)	0 (0%)	/	1.000
Depression	3 (3.13%)	6 (11.1%)	+ 255.6%	0.348
Anxiety	0 (0%)	0 (0%)	/	1.000
Alcohol/drug abuse	7 (7.29%)	6 (11.1%)	+ 52.4%	0.653
**Lombardia**				
Total cases	38,778	21,022	-	-
NPD	128 (0.33%)	140 (0.66%)	+ 100%	0.433
Suicidal ideation	7 (5.47%)	11 (7.86%)	+ 43.7%	0.697
Psychosis	11 (8.59%)	23 (16.4%)	+ 91.2%	0.200
Neurosis	110 (85.9%)	106 (75.7%)	-11.9%	0.945
ED	8 (6.25%)	24 (17.1%)	+ 174.3%	0.748
Depression	0 (0%)	4 (2.86%)	/	0.640
Anxiety	42 (32.8%)	30 (21.4%)	-34.7%	0.063
Alcohol/drug abuse	11 (8.59%)	17 (12.1%)	+ 41.3%	0.562
**Marche**				
Total cases	87,522	51,567	-	-
NPD	376 (0.43%)	231 (0.45%)	+ 4.27%	0.947
Suicidal ideation	1 (0.27%)	0 (0%)	/	0.949
Psychosis	26 (6.92%)	18 (7.79%)	+ 12.7%	0.834
Neurosis	349 (92.8%)	213 (92.2%)	-0.66%	0.884
ED	6 (1.59%)	4 (1.73%)	+ 8.51%	0.974
Depression	0 (0%)	0 (0%)	/	1.000
Anxiety	56 (14.9%)	42 (18.2%)	+ 22.1%	0.432
Alcohol/drug abuse	28 (7.45%)	30 (12.9%)	+ 74.4%	0.185
**Umbria**				
Total cases	7960	4180	-	-
NPD	18 (0.23%)	15 (0.36%)	+ 56.5%	0.889
Suicidal ideation	0 (0%)	0 (0%)	/	1.000
Psychosis	1 (5.56%)	2 (13.3%)	+ 140.0%	0.656
Neurosis	17 (94.4%)	12 (80.0%)	-15.3%	0.409
ED	0 (0%)	0 (0%)	/	1.000
Depression	0 (0%)	0 (0%)	/	1.000
Anxiety	13 (72.2%)	11 (73.3%)	+ 1.53%	0.949
Alcohol/drug abuse	0 (0%)	0 (0%)	/	1.000

During the study period, a total of 533,318 children were admitted to the pED involved in the study. Out of them, 351,559 were admitted before the COVID-19 pandemic, while the remaining 181,759 were admitted during the COVID-19 pandemic. In the pre COVID-19 period, 2369 children were admitted to the pED for any NPD, representing 0.63% of the total admission. In the COVID-19 period, 2242 patients (1.23% of total pED admission) with an NPD entered the pED. Despite a 48.2% decline of pED admissions, there was a significant increase (83.1%) in patient admissions for NPD (*p* < 0.001). Many of these children were affected by eating disorders. The percentage significantly increased in the COVID-19 period as compared to the pre-pandemic study period (8.9 vs 15.9%, *p* < 0.001).

The regions registering a higher number of NPD accesses were Emilia-Romagna (+ 110%), Lazio (+ 107.1%), Lombardia (+ 100%), Friuli-Venezia Giulia (+ 69%), Umbria (+ 56.5%), and Abruzzo (+ 40%).

The most frequent NPD conditions which increased during the COVID-19 pandemic were suicidal ideation (+ 147%), depression (+ 115%), eating disorder (+ 78.4%), and psychosis (+ 17.2%).

The highest increase of pED admissions correlated to suicidal ideation was observed in Lazio (+ 570.5%), Friuli Venezia Giulia (+ 204.8%) and Lombardia (+ 43.7%). As for eating disorder, the greatest increase was recorded in Lombardia (+ 174.3%), Emilia Romagna (+ 113.2%), Friuli Venezia Giulia (+ 59.8%) Lazio (+ 20%) and Marche (+ 8.51%). Admissions to the pED for depression also increased, mainly in Liguria (+ 255.6%), Lazio (+ 63.4%), and Emilia Romagna (+ 6.6%). Psychosis was also a major cause of admission to the pED, with the greatest increase in Umbria (+ 140%), Lombardia (+ 91.2%), Liguria (+ 26.9%) and Friuli Venezia Giulia (+ 20.5%).

The total number of children hospitalized and related NPD diagnoses during the study period are summarized in Table 2.

**Table 2:** Pediatric admissions to pediatric ward and comparison of the two study periods

	PRE	POST	% change	*p-value*
**All pED accesses**	2369	2242	-	-
NPD	599 (25.3%)	833 (37.2%)	+ 47.0%	< 0.001
Suicidal ideation	16 (2.67%)	52 (6.24%)	+ 134%	0.182
Psychosis	176 (29.4%)	217 (26.1%)	-11.3%	0.214
Neurosis	395 (65.4%)	556 (66.7%)	+ 1.22%	0.764
ED	87 (14.5%)	159 (19.1%)	+ 31.4%	0.088
Depression	30 (5.01%)	59 (7.08%)	+ 41.4%	0.439
Anxiety	61 (10.2%)	40 (4.80%)	-52.9%	0.045
Alcohol/drug abuse	42 (7.01%)	74 (8.89%)	+ 26.7%	0.485
**Abruzzo**				
NPD	83 (13.9%)	39 (4.7%)	**-**66.2%	< 0.001
Suicidal ideation	0 (0%)	0 (0%)	/	1.000
Psychosis	16 (19.3%)	9 (23.1%)	+ 19.7%	0.696
Neurosis	55 (66.3%)	26 (66.7%)	+ 0.61%	0.967
ED	11 (13.3%)	2 (5.13%)	-61.3%	0.403
Depression	1 (1.21%)	1 (2.56%)	+ 112.8%	0.889
Anxiety	8 (9.64%)	4 (10.3%)	+ 6.41%	0.949
Alcohol/drug abuse	14 (16.9%)	8 (20.5%)	+ 21.6%	0.707
**Basilicata**				
NPD	24 (4.0%)	18 (2.2%)	-45.0%	0.491
Suicidal ideation	0 (0%)	0 (0%)	/	1.000
Psychosis	10 (41.7%)	12 (66.7%)	+ 60.0%	0.109
Neurosis	14 (58.3%)	6 (33.3%)	-42.9%	0.109
ED	0 (0%)	0 (0%)	/	1.000
Depression	0 (0%)	0 (0%)	/	1.000
Anxiety	12 (50.0%)	2 (11.1%)	-77.8%	0.126
Alcohol/drug abuse	0 (0%)	0 (0%)	/	1.000
**Emilia Romagna**				
NPD	5 (0.8%)	30 (3.6%)	+ 83.3%	0.301
Suicidal ideation	0 (0%)	0 (0%)	/	1.000
Psychosis	2 (40.0%)	3 (10.0%)	-75.0%	0.214
Neurosis	3 (60.0%)	27 (90.0%)	+ 50.0%	0.214
ED	0 (0%)	1 (3.33%)	/	0.890
Depression	0 (0%)	0 (0%)	/	1.000
Anxiety	0 (0%)	0 (0%)	/	1.000
Alcohol/drug abuse	1 (20.0%)	18 (60.0%)	+ 200.0%	0.098
**Friuli Venezia Giulia**				
NPD	85 (14.2%)	87 (10.4%)	**-**26.8%	0.162
Suicidal ideation	2 (2.4%)	10 (11.5%)	+ 388.5%	0.231
Psychosis	26 (30.6%)	16 (18.4%)	-39.9%	0.110
Neurosis	57 (67.1%)	61 (70.1%)	+ 4.56%	0.689
ED	5 (5.89%)	10 (11.5%)	+ 95.4%	0.462
Depression	9 (10.6%)	4 (4.59%)	-56.6%	0.432
Anxiety	21 (24.7%)	15 (17.2%)	-30.2%	0.328
Alcohol/drug abuse	5 (5.88%)	8 (9.19%)	+ 56.3%	0.664
**Lazio**				
NPD	258 (43.1%)	443 (53.2%)	+ 23.4%	< 0.001
Suicidal ideation	2 (0.78%)	19 (4.29%)	+ 453.3%	0.369
Psychosis	87 (33.7%)	124 (27.9%)	-16.9%	0.143
Neurosis	169 (65.5%)	297 (67.0%)	+ 2.35%	0.694
ED	56 (21.7%)	111 (25.1%)	+ 15.4%	0.392
Depression	18 (6.97%)	47 (10.6%)	+ 52.1%	0.354
Anxiety	8 (3.10%)	10 (2.26%)	-27.2%	0.829
Alcohol/drug abuse	6 (2.33%)	15 (3.39%)	+ 45.6%	0.785
**Liguria**				
NPD	46 (7.7%)	80 (9.6%)	+ 24.7%	0.473
Suicidal ideation	0 (0%)	0 (0%)	/	1.000
Psychosis	14 (30.4%)	33 (41.3%)	+ 35.5%	0.242
Neurosis	32 (69.6%)	46 (57.5%)	-17.3%	0.192
ED	1 (2.17%)	0 (0%)	/	1.000
Depression	2 (4.35%)	4 (5.00%)	+ 15.0%	0.944
Anxiety	0 (0%)	0 (0%)	/	1.000
Alcohol/drug abuse	9 (19.6%)	21 (26.6%)	+ 34.2%	0.470
**Lombardia**				
NPD	24 (4.0%)	36 (4.3%)	+ 7.5**%**	0.906
Suicidal ideation	6 (25.0%)	11 (30.6%)	+ 22.2%	0.673
Psychosis	5 (20.8%)	10 (27.8%)	+ 33.3%	0.598
Neurosis	13 (54.2%)	15 (41.7%)	-23.1%	0.328
ED	2 (8.33%)	9 (25.0%)	+ 200.0%	0.206
Depression	0 (0%)	3 (8.33%)	/	0.527
Anxiety	2 (8.33%)	1 (2.78%)	-66.7%	0.673
Alcohol/drug abuse	3 (12.5%)	1 (2.78%)	-77.8%	0.461
**Marche**				
NPD	29 (4.8%)	22 (2.6%)	**-**45.8%	0.411
Suicidal ideation	0 (0%)	0 (0%)	/	1.000
Psychosis	13 (44.8%)	7 (31.8%)	-29.0%	0.357
Neurosis	16 (55.2%)	15 (68.2%)	+ 23.6%	0.357
ED	1 (3.45)	0 (0%)	/	1.000
Depression	0 (0%)	0 (0%)	/	1.000
Anxiety	7 (24.1%)	2 (9.09%)	-62.3%	0.287
Alcohol/drug abuse	3 (10.3%)	1 (4.54%)	-56.1%	0.682
**Umbria**				
NPD	45 (7.5%)	78 (9.4%)	+ 25.3%	0.490
Suicidal ideation	6 (13.3%)	12 (15.4%)	+ 15.8%	0.827
Psychosis	3 (6.67%)	3 (3.85%)	-42.3%	0.763
Neurosis	36 (80.0%)	63 (80.8%)	+ 0.36%	0.935
ED	11 (24.4%)	26 (33.3%)	+ 36.4%	0.342
Depression	0 (0%)	0 (0%)	/	1.000
Anxiety	3 (6.67%)	6 (7.69%)	+ 15.3%	0.913
Alcohol/drug abuse	1 (2.22%)	2 (2.56%)	+ 15.3%	0.971

The total number of patients admitted for NPD before the pandemic was 599 of 2369 admissions (25.3%). During the pandemic, the total number was 833 of 2242 (37.1%) documenting a 39.5% increase in NPD hospitalizations during the study period.

The regions with the greatest increase in NPD hospitalizations were Emilia-Romagna (+ 83.3%), Umbria (+ 25.3%), and Liguria (+ 24.7%).

The NPD disorders that required hospitalizations were suicidal ideation (+ 134%), depression (+ 41.4%), eating disorder (+ 31.4%), and drug abuse (+ 26.7%). In detail, the greatest increase in hospitalizations for suicidal ideation was observed in Lazio (+ 453.3%), Friuli Venezia Giulia (+ 379.2%), Lombardia (+ 22.2%) and Umbria (+ 15.4%).

Regarding hospitalizations for eating disorders, the greatest increase was registered in Lombardia (+ 200%), Friuli Venezia Giulia (+ 95.4%), Umbria (+ 36.4%) and Lazio (+ 15.4%). Hospitalizations for depression were also implemented, especially in Abruzzo (+ 112.8%), Lazio (+ 52.1%) and Liguria (+ 15%). Drug-related hospitalizations also increased, especially in Emilia Romagna (+ 200%), Friuli Venezia Giulia (+ 56.3%), Lazio (+ 45.6%), Liguria (+ 34.2%), Abruzzo (+ 21.6%), and Umbria (+ 15.3).

## Discussion

The COVID-19 pandemic had a major impact on children's health. Although the percentage of children hospitalized was minimal compared to adults, the consequences on their neuropsychological development were extremely severe [6, 7]. Our study showed an overall increase of 83.1% and 39.5% for NPD admissions for pED access and pediatric wards admissions, respectively, during the COVID-19 pandemic. Thus, despite reduction in children admission to pediatric hospitals, likely due to fear of COVID-19 and reduced spread of other common infections, the need for NPD help was so pressing that Italian families were persuaded to reach out for pED. Other reports confirmed our findings, emphasizing the neuropsychological impairment that young people are facing [9, 10]. For example, despite an absolute reduction of weekly visits for mental health conditions (MHCs) during the COVID-10 pandemic, the US CDC reported a relative increase of visits for overall MHCs compared to all pediatric visits in the same period [9]. An earlier report indicated the same trend, with more frequent involvement of white females over the age of 12 [10].

Thus, while school closures were initially a valuable tool to protect children from infection at the onset of the pandemic, they ultimately produced a negative impact on their mental health. Indeed, building, maintaining, and enjoying social connections with peers plays a crucial role in the development of mental health during childhood and adolescence [11].

Over the study period, suicidal ideation (+ 147%), depression (+ 115%), eating disorders (+ 78.4%), and psychosis (+ 17.2%) were the most observed NPDs during the COVID-19 pandemic in children. According to our data, the admission of suicidal ideation in pEDs increased dramatically after the onset of COVID-19 in children (+ 147%). Suicidal ideation is closely related to stressful events, such as pandemic fear and social isolation of vulnerable individuals [12–14].

Among the risk factors for suicidal ideation, eating disorders have been specifically identified [15, 16].

Of note, a 78.4% increase in eating disorders in children was observed on admission to pED, consistent with recent reports [17]. Daily life restrictions, stress, and exposure to social media have been commonly identified as predictors of the onset of eating disorders in youth during the first wave of COVID 19 [17, 18].

Of note, in COVID-19 affected people, a prolonged sense of smell and tasting may potentially contribute to a decreased appetite [19, 20].

Although social media and smartphones have been crucial for children and adolescents to communicate during restraints, pathological use has been widely linked with promoting self-injurious behaviors and eating disorders. Platforms such as “Tik Tok “ have recently been blamed for the spread of pro-anorexia, pro-suicide, and self-harm videos [21–23].

In addition, a large percentage of websites (56%) such as Twitter or Instagram accounts promote “pro-anorexia” content, being particularly followed by adolescents aged 13–17 years [24–26].

An increased occurrence of depression and anxiety during the pandemic has been confirmed by previous reports [3, 27]. A recent meta-analysis demonstrated that approximately 25% and 20% of children globally experienced clinically elevated depression and anxiety symptoms, respectively, in the first year of the COVID-19 pandemic. The authors also showed that these estimates were twice as high as pre-pandemic values [28].

Finally, our study highlights an increase in pediatric NPD hospitalizations during the COVID-19 pandemic (+ 39.5%), mainly for suicidal ideation (+ 134%), depression (+ 41.4%), eating disorders (+ 31.4%), and drug abuse (+ 26.7%). This result is noteworthy as it highlights the seriousness of the situation. Indeed, daily treatment was not sufficient to address the situation for affected children.

Future studies are needed to investigate the long-term consequences of increased NPD in childhood.

For now, it is important to highlight the existence of a “Second Pandemic,” which did not affect the body, but primarily the brain of vulnerable individuals. Parent-reported mental health problems were more likely to affect children with low socioeconomic status, complex chronic diseases, and those whose parents tested positive for depression [29].

Appropriate services should be made available for people in crisis and those with new or existing mental health problems. Considering that child and adolescent mental health services were already overburdened and underfunded before the onset of the pandemic, there is an urgent need for government investment to support and improve existing services and youth mental health research [30]. It is critical to emphasize early identification of signs that may be related to an NPD, such as reduced interest in food, reduced time spent with peers, changes in behavior, and close association with social media. Neuropsychological assessment should be pushed at the primary level, in the pediatrician's office, to facilitate early capture of the sign of impairment, emphasizing social-relational dimensions and affective-behavioral skills. The aim of the assessment is to ascertain the need for an individualized program to prevent or address difficulties that have arisen during the COVID-19 period. When necessary, with the use of validated instruments capable of recognizing potentially critical situations that may require second level assessments. Integration and connections in individual district areas between health services, schools, support services and social services, through the work of psychologists, educators and community nurses should be developed.

A limit of the study is that we clinical characteristic of patients can not be examined as it was based on ICD classification. Further researches may focus on detailed aspects.

## Conclusion

This is the first study proposed by the Italian Pediatric Society that provides a complete numerical description of the phenomenon of neuropsychiatric disorders in children and adolescents during the COVID-19 pandemic, emphasizing the negative influence of lockdown on children's psychopathological behavior. The Italian Society of Pediatrics recognizes the fundamental role of the pediatrician in health promotion and social-emotional development of children, through the creation of collaborative relationships between children, families, schools, and all specialists involved in supporting children's mental health.

## Supplementary Information

**Additional file1: Table S1.** Regional centers enrolled in the study, list of hospitals and departments involved in the study.

## Data Availability

The datasets used and/or analysed during the current study are available from the corresponding author on reasonable request.

## Abbreviations

SIP: Italian Pediatric Society
pED: Pediatric Emergency Departments
NPD: Neuropsychological disorders
ICD: International Classification Codes
