# Supplementary material for: The pandemic within the pandemic: the surge of neuropsychological disorders in Italian children during the COVID-19 era
Source: Ital J Pediatr. 2022 Jul 27;48:126. doi: 10.1186/s13052-022-01324-4 (PMC9326438; doi:10.1186/s13052-022-01324-4)
Supplement: Supplementary file 1 — Additional file1: Table S1. Regional centers enrolled in the study, list of hospitals and departments involved in the study. [file 13052_2022_1324_MOESM1_ESM.docx]

Supplementary Table1. Regional centers enrolled in the study, list of hospitals and departments involved in the study

| **Region** | **Italian Province** | **Hospital/department** |
| --- | --- | --- |
| Abruzzo | Chieti | Clinica Pediatrica Ospedale SS Annunziata |
|  | L’Aquila | Clinica Pediatrica Università dell’Aquila |
|  | Pescara | Pediatria, Ospedale Spirito Santo |
| Basilicata | Potenza | Ospedale San Carlo |
| Emilia-Romagna | Bologna | Ospedale S. Orsola  Ospedale Maggre |
|  | Cesena | Ospedale Bufalini |
|  | Forlì | Ospedale Morgagni-Pierantoni |
|  | Piacenza | Ospedale di Piacenza |
|  | Ravenna | Ospedale Santa Maria delle Croci |
|  | Rimini | Ospedale Infermi |
| Friuli-Venezia Giulia | Gorizia | Ospedale Gorizia-Monfalcone |
|  | Pordenone | Ospedale di Pordenone |
|  | Trieste | Ospedale Burlo Garofolo |
|  | Udine | Ospedale di Udine  Ospedale Latisana-Palmanova |
| Lazio | Roma | Ospedale Pediatrico Bambino Gesù |
| Liguria | Genova | Ospedale Gaslini |
| Lombardia | Brescia | Clinica Pediatrica di Brescia |
|  | Milano | Policlinico di Milano |
| Marche | Pesaro e Urbino | Ospedali Riuniti Marche Nord |
| Umbria | Città di Castello | Ospedale di Città di Castello |
|  | Foligno | Ospedale di Foligno |
|  | Orvieto | Ospedale di Orvieto |
|  | Perugia | Ospedale di Perugia |
|  | Terni | Ospedale di Terni |
